# Manufacture of Bioplastics Prepared from Chitosan Functionalized with *Callistemon citrinus* Extract

**DOI:** 10.3390/polym16192693

**Published:** 2024-09-24

**Authors:** Marika Avitabile, Seyedeh Fatemeh Mirpoor, Sefora Esposito, Giusi Merola, Loredana Mariniello, Giuseppe Tancredi Patanè, Davide Barreca, Concetta Valeria Lucia Giosafatto

**Affiliations:** 1 Department of Chemical Sciences, University of Naples Federico II, 80126 Naples, Italy; marika.avitabile@unina.it (M.A.); sefora.esposito@unina.it (S.E.); giusi.merola@studenti.unina.it (G.M.); loremari@unina.it (L.M.); 2 Department of Food and Nutritional Sciences, University of Reading, P.O. Box 226, Whiteknights, Reading RG6 6AP, UK; s.mirpoor@reading.ac.uk; 3 Department of Chemical, Biological, Pharmaceutical and Environmental Science, University of Messina, 98166 Messina, Italy; giuseppe.patane@studenti.unime.it (G.T.P.); davide.barreca@unime.it (D.B.)

**Keywords:** *Callistemon citrinus*, chitosan, antioxidant properties, film mechanical properties, barrier properties

## Abstract

The exploration of natural resources in bioplastics has advanced the development of bio-based materials. Utilizing the casting, chitosan (CH)-based films were manufactured with different glycerol (GLY) percentages (from 0 to 50% *w*/*w* of CH) and anthocyanin-enriched fractions (from 0 to 5% of *w*/*w* CH) of acidified ethanol extract of *Callistemon citrinus* flowers (CCE). *Callistemon citrinus* is an ornamental plant known for its bioactive compounds endowed with health benefits. The hydrocolloid films showed promising mechanical properties. The 30% GLY + 5% CCE film achieved an elongation at break of 57.4%, comparable to the 50% GLY film while possessing enhanced tensile strength and Young’s modulus. The CCE, rich in antioxidants, acted as a plasticizer, improving films’ flexibility and manageability. The films exhibit hydrophilic characteristics with moisture content and uptake values reflecting their water-absorbing capacity, while films with 30% GLY and 5% CCE exhibit enhanced hydrophobicity. In addition, CCE characterization reveals significant polyphenol content (734.45 mg GAE/g), highlighting its antioxidant capacity. Moreover, CCE supplies remarkable antioxidant properties to the films. These findings suggest the potential of these bioplastics for industrial applications as a sustainable solution to traditional plastics and in reducing environmental impact while preventing oxidative reactions in packaged products.

## 1. Introduction

The pressure from the conventional plastics market has driven research toward identifying renewable resources for bioplastics, such as plant-based polysaccharides, proteins, and bioactive compounds, for the development of bioplastics [1]. These alternatives help to decrease dependence on fossil fuels while offering improved biodegradability and additional functional benefits, such as antioxidant or antimicrobial properties [2]. These features are particularly advantageous in food packaging, where preserving product quality and extending shelf life are crucial [3]. As a result, there is a growing demand for innovative solutions in food packaging that effectively balance functionality with environmental sustainability [4].

The utilization of flower extracts in bioplastics represents a promising and innovative advancement in this field [5]. Flowers, rich in bioactive compounds, offer potential advantages such as enhanced biodegradability and improved material properties [6]. By adding these natural extracts, bioplastics can achieve both sustainability and functionality, addressing the pressing need for environmentally friendly solutions while maintaining the performance qualities of traditional plastic [7]. Hydrocolloid films, derived from biopolymers in diverse waste sources, represent an innovative and eco-friendly approach [1]. Primarily used in food wrapping, these advanced bioplastics based on polysaccharides and proteins aim to limit moisture, gases, microbes, and solutes, thus preventing product spoilage [8].

**Figure 1:**
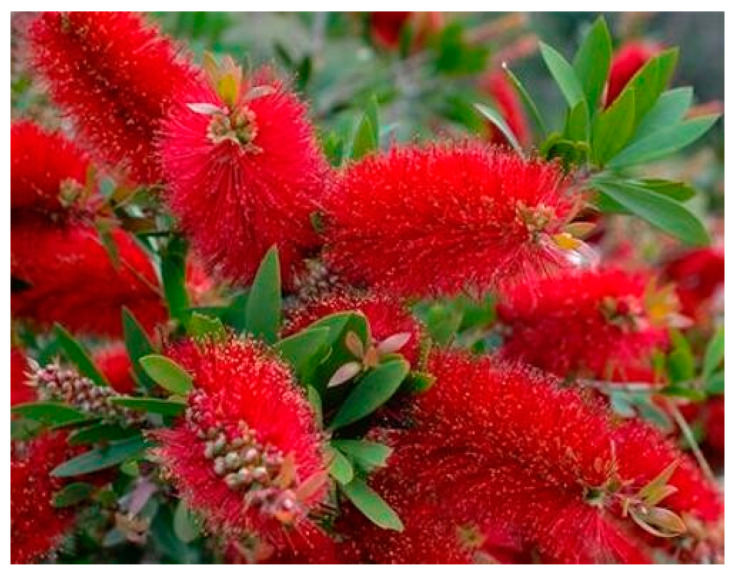
*Callistemon citrinus* flowers.

This allowed for a comparison of the hydrocolloid films against traditional plastics, such as low-density polyethylene (LDPE), which is commonly used as a standard due to its widespread use and well-established performance characteristics [9]. LDPE, a thermoplastic polymer, is characterized by its highly branched molecular structure, which results in lower crystallinity and weaker intermolecular forces compared to more rigid polymers. This structure gives LDPE its notable flexibility, making it a versatile material in a range of applications. The mechanical properties of LDPE, such as its high elongation at break (349%) and moderate tensile strength (9.93 MPa), set a benchmark for evaluating the performance of bioplastics [10]. Nevertheless, the mechanical strength of many bioplastics does not match up to synthetic packaging such as LDPE [4,11]. For instance, chitosan films with 30% GLY showed different mechanical properties. Despite the addition of GLY, which enhances flexibility, these films achieved an elongation of only 35% and a tensile strength of 15.29 MPa. Although the tensile strength of the chitosan films is higher than that of LDPE, the overall mechanical performance still falls short when compared to synthetic packaging standards. This comparison underscores the challenges bioplastics face in meeting the mechanical performance levels of traditional synthetic materials, while also illustrating how various polymer structures and additives affect the properties of these films [12]. Methods and technologies have been used to enhance the properties of the polymers, often by adding plasticizers and nanoparticles to boost film flexibility and decrease brittleness [13]. GLY, among other polyhydric alcohols, is a widely used plasticizer in this field. Its advantages—being less sweet, cost-effective, and FDA-approved—make it particularly effective [14]. GLY integrates into protein networks, forming hydrogen bonds that reduce interactions between proteins and modify the polymer’s structure, making it more porous, flexible, and less cohesive. A continuing issue with this approach is the tendency for glycerol to leach from the film over time. In the literature, the method of internal plasticization proves more effective than external plasticization, offering greater flexibility and strength, and it eliminates issues related to plasticizer leaching. For example, trans-esterification with vinyl laurate was used to internally plasticize hemicellulose, resulting in a roughly 200% increase in its elongation [15]. Sorbitol is another plasticizer that enhances flexibility similarly to glycerol; xylitol shares similarities with sorbitol, and it offers additional antimicrobial properties [16]. Various glycols, such as polyethylene glycol, are also used as plasticizers, but they can influence the tensile strength of the film. Natural sugars are used in some biopolymeric films as plasticizers, but their effectiveness can vary depending on the specific type of sugar used, and while they generally have good compatibility with biopolymeric materials, they may affect the mechanical properties differently compared to other plasticizers [17]. The studies indicated that a plasticizer concentration of 20% with glycerol appears to be adequate to produce a flexible chitosan film with good stability for up to 5 months of storage [17]. In addition to increasing the amount of plasticizer in blended films, the elongation also increased. However, at higher plasticizer concentrations, both the tensile strength and modulus were found to decrease [16]. This versatility makes GLY a top choice for improving film properties [18], highlighting that factors such as the film matrix and the type and quantity of plasticizer used are critical considerations when applying chitosan for edible films and coatings. Second only to cellulose in abundance, chitin is commonly transformed into chitosan at an industrial scale. Chitosan (CH), derived from the N-deacetylation of chitin, is a linear polymer characterized by 2-amino-2-deoxy-D-glucopyranose units connected via (1→4) linkages with some residual D-glucosamine. Unlike chitin, CH is more valuable for industrial applications due to its amino and hydroxyl groups, which are highly reactive, its reduced crystallinity that enhances its reactivity with chemicals, and its ability to dissolve in organic acidic solutions with a pKa of 6.5 [19]. CH features a degree of deacetylation of 75–85%, a molecular weight between 50,000 and 190,000 Da, and a viscosity of 20–300 cP when dissolved at 1 wt.% in 1% acetic acid at 25 °C (measured with a Brookfield viscometer). It is soluble in dilute aqueous acids, making it versatile for various industrial applications. *Callistemon citinus* (Figure 1) is a very common ornamental plant worldwide, about which there has been an increased interest in recent years as an abundant and affordable natural resource of anthocyanin compounds [20,21]. The plant chemical analysis revealed that different parts of the plant contain polyphenols, such as alkaloids, monoterpenoids, aliphatic acids, tannins, sesquiterpenes, triterpenoids, and steroids [20,21]. This phytocomplex, particularly rich in polyphenols, explains why this plant has always been used for therapeutic purposes. In the literature, it has already been reported that this plant is used to treat various inflammation-based disorders, but also for its important antimicrobial activity [22]. In addition to what has already been mentioned [21] demonstrated that this phytocomplex has important anti-angiogenic, antioxidant, and cytoprotective activity. Studies have already shown the promising effects of *Callistemon citrinus* flower extract on food preservation [20]. In this work, it was decided to incorporate a fraction with high anthocyanin content from the acidified ethanol extract of *Callistemon citrinus* flowers (CCE) into a CH-based film for potential use in the food packaging industry.

## 2. Material and Methods

### 2.1. Materials

Methanol, 2,2-diphenyl-1-picrylhydrazyl (DPPH), acetic acid, potassium peroxydisulfate, 2,2′-azino-bis (3-ethylbenzothiazoline-6-sulfonic acid) diammonium salt (ABTS^•+^), 2,4,6-Tris(2-pyridyl)-S-triazine (TPTZ), ethylenediaminetetraacetic acid (EDTA), sodium phosphate dibasic, potassium phosphate monobasic, sodium acetate, and 6-hydroxy-2,5,7,8-tetramethylchromane-2-carboxyl acid (Trolox) were purchased from Merck (Darmstadt, Germany). Low molecular weight (50,000–190,000 Da) CH from shrimp waste was supplied by Sigma Aldrich (St Louis, MO, USA) and GLY from Carlo Erba (Milan, Italy). Reagents and the rest of the chemicals used and mentioned in the study are from Steinheim (Germany). Folin–Ciocalteu (FC) reagent, gallic acid, sodium carbonate, and absolute methanol were from Merk (Darmstadt, Germany). All other chemicals and solvents utilized in this study were of analytical grade, unless otherwise noted.

### 2.2. Methods

#### 2.2.1. Enriched Fraction of Acidified Ethanol Extract of *Callistemon citrinus* Flowers (CCE) Preparation and Characterization

##### Preparation of Fortified Extract of *Callistemon citrinus* Flowers (CCE)

Flowers of *C. citrinus* were collected from local nurseries in Messina (Italy), air-dried until their humidity content was lower than 2% dry matter, ground into powder using a mortar, and then used for anthocyanin extraction according to [21]) with some modifications. Briefly, 10.0 g of powder of *C. citrinus* flowers was extracted using a mixture of acetic acid:ethanol:water (1:70:29, *v*/*v*/*v*) at a ratio of 1:10 (*w*/*v*), several folds (at least three times), with multiple extractions performed until the flowers were fully depleted of anthocyanins. This mixture was selected through an analysis of the literature because it is suitable for the extraction of anthocyanins. The extracted solution was concentrated to 10.0 mL with a rotary evaporator and further processed through solid-phase extraction using a Supelclean™ LC-18 SPE cartridge (Supelco Ltd., Bellefonte, PA, USA). The final anthocyanin-enriched fraction was eluted with an ethanol (1:70:29, *v*/*v*/*v*) mixture, then evaporated to dryness in a vacuum desiccator. The resulting powder was stored in the dark at 4 °C. Aliquots of this powder were used for further analyses and indicated as CCE.

##### CCE Profile Characterization by Reverse Phase High-Performance Liquid Chromatography Coupled with Diode Array and Electrospray Mass Spectrometry Detection (RP-HPLC-DAD-ESI-MS/MS) Analysis

The qualitative and quantitative analysis of compounds present in CCE was performed utilizing an ThermoQuest Model LCQ-Duo, which featured a diode array spectrophotometer and an ion trap mass spectrometer with an electrospray ionization source. The chromatographic separation was carried out using a Discovery C18 column (150 mm × 4.6 mm, 5 μm particle size; Supelco Ltd., Bellefonte, PA, USA) with a pre-column Discovery^®^ C18 Supelguard^™^ (20 mm × 4 mm, 5 μm particle size; Supelco Ltd., Bellefonte, PA, USA) and a mobile phase consisting of solvent A (formic acid 0.1%) and solvent B (acetonitrile), following an gradient elution program of: 0–3 min, 0% B; 3–9 min, 3% B; 9–24 min, 12% B; 24–30 min, 20% B; 30–33 min, 20% B; 33–43 min, 30% B; 43–63 min, 50% B; 63–66 min, 50% B; 66–76 min, 60% B; 76–81 min, 60% B; 81–86 min, 0% B, with a 4-min equilibration period, for a total run time of 90 min. The flow rate was 0.4 mL/min, the injection volume was 5 µL, and the column temperature was 25 °C. The UV-Vis spectra of anthocyanins were recorded from 190 to 600 nm, and chromatograms were obtained at 260, 292, 330, 370, and 520 nm to recognize all classes of polyphenols. Nitrogen was utilized as a sheath gas with a stream of 50 arbitrary units. The mass spectrometer settings included a capillary temperature of 250 °C, a spray needle voltage of 4.50 kV, and ESI capillary voltages of +3 and −47 V for positive and negative polarities, respectively. Tube lens offsets were set at 0 V and −25 V for positive and negative polarities, respectively. Compound identification was confirmed by comparing retention times (Rt), UV-Vis, and mass spectra with those reported in the literature and comparison with commercially available authentic standards. Quantification was expressed as cyanidin-3-O-glucoside equivalents/100 g of dry extract (DE) by using an external calibration curve of the reference standard.

##### Determination of Total Phenolic Content

Determination of the total phenolic content of CCE was performed according to the method of Singleton et al. and adapted for 96-well microplates [23,24]. Gallic acid, prepared in four concentrations ranging from 5 to 50 μg/mL, was used as a standard to obtain a gallic acid calibration curve. Various samples of gallic acid were prepared in methanol, while the blank was prepared using methanol only. Extracts were used in concentrations (0.06, 0.125, 0.25, 0.5, and 1.0 mg/mL); 0.75 g of sodium carbonate (Na_2_CO_3_) in a 20% aqueous solution was added to make the environment basic, and 0.5 mL of Folin-Ciocalteu reagent was added. The prepared samples were gently stirred and incubated in the dark at room temperature for 1 h before conducting the spectrophotometric measurement of the samples at 760 nm. The total phenolic concentration was determined by comparison with the standard calibration curve of gallic acid. All tests were carried out in triplicate, and the results are presented as mean values. The total phenolic content was expressed as milligrams of gallic acid equivalents (GAE) per gram of dry extract (DM).

##### DPPH Radical Scavenging Activity

Ten microliters of each sample (with the starting concentrations of CCE and bioplastic from 1 mg/mL) were combined to 100 μL of 67.5 μmol/L DPPH solution in methanol, and the mixture was diluted with an additional 190 μL of methanol. For the controls, 10 μL of the sample was replaced with distilled water. In blank probes, methanol (290 μL) and each sample (10 μL) were mixed, while in the blank probe for the control, only 300 μL of methanol was added.

Absorbance measurements were recorded at 515 nm after 1 h. All samples and the control were prepared in triplicate. The percentage of inhibition achieved by different concentrations of samples in the antioxidant assays performed was calculated using the following equation:

I (%) = (A_0_ − A)/A_0_ × 100 (1)

where A_0_ was the absorbance of the control response and A was the absorbance of the inspected samples, with both adjusted for the value of the corresponding blank probes. Inhibition-concentration curves were plotted using Origin software, version 8.0, and IC50 values (concentration of extract required to inhibit 50% of DPPH formation) were determined. Each assay result was reported as the mean ± standard deviation (SD) of three measurements [24].

#### 2.2.2. Film-Forming Solutions (FFSs) Preparation and Derived Films Characterization

##### Zeta Potential and Particle Size Measurements of the Film Forming Solutions (FFSs)

The effect of incorporating CCE into FFSs was analyzed by evaluating surface charge potential and particle size distribution. Each FFS was diluted to a concentration of 1 mg/mL for this test. Zeta potential and particle dimension values were measured with a Zetasizer Nano-ZSP (Malvern^®^, Worcestershire, UK) with a 633 nm wavelength and a 4 mW helium-neon laser. The analysis was carried out at a temperature of 25 °C with an applied voltage of 200 mV, and each measurement took approximately 10 min. Zeta potential was determined using the instrument’s Zetasizer software version 7.12 using the electrophoretic mobility at 200 mV, utilizing the Henry equation. The software also calculated the average particle diameter using dynamic light scattering and the polydispersity index (PI), which indicates the relative variation in particle size distribution. Zeta potential and particle size measurements were conducted in triplicate, and all the results were reported as mean ± standard deviations [25]. A stock solution was prepared by dissolving CH 2% (*w*/*v*) in HCl 0.1 N and stirring overnight. The pH was adjusted to pH 2. FFSs were formulated by using increasing concentrations of GLY 50 mg/mL (10%, 20%, 30%, and 50% *w*/*w* of CH) added immediately before casting. The final volume of all samples was adjusted to 115 mL with distilled water and buffered to maintain pH 2, and they were then stirred for 10 min. Then, 1 mL aliquot was taken from each sample for zeta potential and particle size analysis.

##### Casting of the Film-Forming Solutions (FFSs)

The FFS (115 mL) was poured onto the Coatmaster and left to air dry for 16 h. In a second analysis, FFSs were prepared by adding different concentrations of CCE (1.25%, 2.5%, 5% *w*/*w* of CH) dissolved in water into the 30% GLY (*w*/*w* of CH). The FFSs were stirred for 10 min before casting. All samples were adjusted to a final volume of 115 mL.

##### Film Mechanical Properties and Sealing Strength

Tensile strength (TS), elongation at break (EB), Young’s modulus (YM), and sealing strength (SS) were measured by using an Instron Universal Testing Instrument (Instron Engineering Corp., Canton, MA, USA, mode l5543A) according to ASTM (1997) [26] and following the procedures described [27,28]. Before being tested, all dried films were conditioned at 25 °C and 50% of R.H. for 24 h by placing them into a desiccator in which there was a saturated Mg(NO_3_)_2_ solution. Film samples were cut into strips measuring 10 mm wide and 80 mm in length using a precision razor blade. Testing was performed on three or more strips for each film type. Each film strip was placed between the pneumatic jaws of the Instron, which were adjusted to an initial gauge of 40 mm and then stretched at a rate of 10 mm/min until the sample failed.

To test SS, the film samples were cut into strips of 5 × 2.5 cm; one strip was placed on top of another one to be sealed. Those two strips were sealed by a heat sealer (Magic Vac^®^ Axolute, mod: P0608ED by Flaem Nuova S.p.A., Brescia, Italy). The sealing parameters were studied according to [29], and the SS (N/m) was calculated as the following equation:

SS (N/m) = Peak force/film width(2)

The sealing test is critical for packaged products, as it ensures the integrity and protection of the items. The ability to achieve sealing is essential in the food packaging industry.

##### Film Moisture Content and Uptake

Film moisture content analysis was carried out by measuring the weight loss of each film after 24 h in an oven at 105 °C. After drying, the samples were weighed, and the moisture content was calculated as:

Moisture content (%) = [(Wi − Wf)/Wi] × 100(3)

Wi is the initial weight of the film and Wf is the film weight after drying [25].

To evaluate film moisture absorption, the mass of each film was recorded after it had been dried at 105 °C for 24 h and after a further 24 h in a conditioning environment at 50% RH over a saturated solution of Mg(NO_3_)_2_ [25]. The moisture absorption was determined using the equation:

Moisture uptake (%) = [(Ws − Wi)/Ws] × 100(4)

Wi is the weight of dried films and Ws is the weight of the swollen film (kept in a glass chamber with saturated Mg(NO_3_)_2_, with constant conditions at 50% RH). All the experiments were performed in triplicate.

##### Film Water Contact Angle (WCA)

The contact angle between the water and CH-based films was measured by using a contact angle goniometer. Five droplets (10 μL) of distilled water were placed on both surfaces of each film at different points, and the images were captured at the moment that the drop was in contact with the film surface. The mean value of the contact angle was determined with ImageJ software 1.53t [25].

##### Film Barrier Properties

The permeability of the films to water vapor (WV), oxygen (O_2_), and carbon dioxide (CO_2_) was evaluated in duplicate for each sample, following the modified ASTM methods D3985-05 (2010) [30], ASTM F2476-05 (2005) [31], and ASTM F1249-13 (2013) [32] using a MultiPerm instrument (ExtraSolutions s.r.l., Pisa, Italy). The film samples were conditioned for 24 h at 50% relative humidity (RH) before being placed into the aluminum masks, reducing their exposed surface area to 2 cm^2^. Low-density polyethylene (LD-PE) was used as the reference material.

##### Fourier Transform Infrared Spectroscopy (FTIR-ATR)

FTIR spectra were recorded using a Perkin Elmer spectrum in the range of 4000–400 cm^−1^ equipped with a universal ATR (attenuated total reflection) sampling accessory. All the samples were analyzed at room temperature.

##### Film Antioxidant Properties

###### DPPH Assay

A method utilizing the stable free radical 2,2-diphenyl-1-picrylhydrazyl (DPPH•) was performed according to the technique [33]. A 1 cm^2^ piece of each film was mixed with 80 μM DPPH• in methanol in a final volume of 1 mL. Absorbance changes at 517 nm were recorded over a 30-min period using a Varian Cary 50 UV-Vis spectrophotometer. The DPPH radical concentration in the cuvette (with a 1.0 cm path length) was selected to ensure absorbance values were below 1.0. The percentage inhibition of radical scavenging activity was calculated using the following equation:

I (%) = [(Ac − As)/Ac] × 100(5)

where Ac is the absorbance of the control and As is the absorbance of the sample. All tests were run in triplicate, and the results were expressed as means ± standard deviation (SD).

##### ABTS Radical Scavenging Assay

The free radical scavenging activity using 2,2′-azino-bis(3-ethylbenzothiazoline-6-sulphonic) acid (ABTS) was assessed using a decolorization assay [21]. Each 1 cm^2^ film sample was treated with the ABTS•+ radical cation, and the absorbance at 734 nm was recorded with a spectrophotometer after 6 min.

##### Ferric-Reducing Antioxidant Power (FRAP)

The FRAP assay was performed according to [33]. A fresh working FRAP reagent was prepared daily by mixing 25 mL of acetate buffer (300 mM, pH 3.6), 2.5 mL of 2,4,6-Tris(2-pyridyl)-S-triazine (TPTZ) solution (10 mM in 40 mM HCl), and 2.5 mL of FeCl3 (20 mM). The reagent was heated to 37 °C, and 1500 μL of it was transferred to a cuvette (1.0 cm path length), where the initial absorbance was recorded. A 1 cm^2^ piece of each film was then added to the cuvette, and the absorbance at 593 nm was measured after 4 min using a Varian Cary 50 UV-Vis spectrophotometer. All tests were performed in triplicate, and the results were reported as means ± standard deviation (SD).

##### Total Antioxidant Capacity Assay (TAC)

The assay was performed using a commercial Total Antioxidant Capacity Assay Kit made available by Merk (Darmstadt, Germany), following the instructions of the supplier.

#### 2.2.3. Statistical Analysis

All statistical analyses were carried out using SPSS software (version 29, SPSS Inc., Chicago, IL, USA). To identify significant differences among the samples, one-way analysis of variance (ANOVA) and Duncan’s multiple range tests were used, with a significance level set at *p* < 0.05. All treatments were tested in triplicate.

## 3. Results

### 3.1. Preparation, Identification, and Quantification of CCE by RP-HPLC-DAD-ESI-MS/MS

The preparation of the CCE, based on a protocol developed over the years [21], led us to obtain it with a yield of ~22 ± 1.8%, starting with dried flowers with a moisture lower than 2%. For the first and preliminary characterization, we analyzed the total phenolic content in CCE using the Folin-Ciocalteu reagent. Gallic acid allowed us to express the polyphenol content as milligrams of gallic acid equivalent per gram of extract powder. The spectrophotometric measurement of the samples showed that the extract had a polyphenol amount of 734.45 ± 61.8 mg GAE/g. The data obtained from this experiment are comparable to what has been reported in the literature under the same conditions [21]. The main categories and typical elements of polyphenolic compounds found in the examined CCE were further investigated using RP-HPLC-DAD-MS/MS. They were identified based on their retention time, UV spectra, and MS and MS/MS data and compared to a standard sample and the existing literature [8]. The chromatographic separation of the CCE, recorded at 517 nm, showed the presence of only four peaks, according to recent literature data [21], with the principal classes of polyphenolic compounds detected and including mainly anthocyanins. The identified compounds are depicted in Table 1, with their Rt and quantifications. The inspection of UV-visible spectra and MS data at the other recorded wavelengths shows the absence of other compounds in the CCE.

### 3.2. DPPH Radical Scavenging Activity of Callistemon citrinus Flower Extract

**Figure 2:**
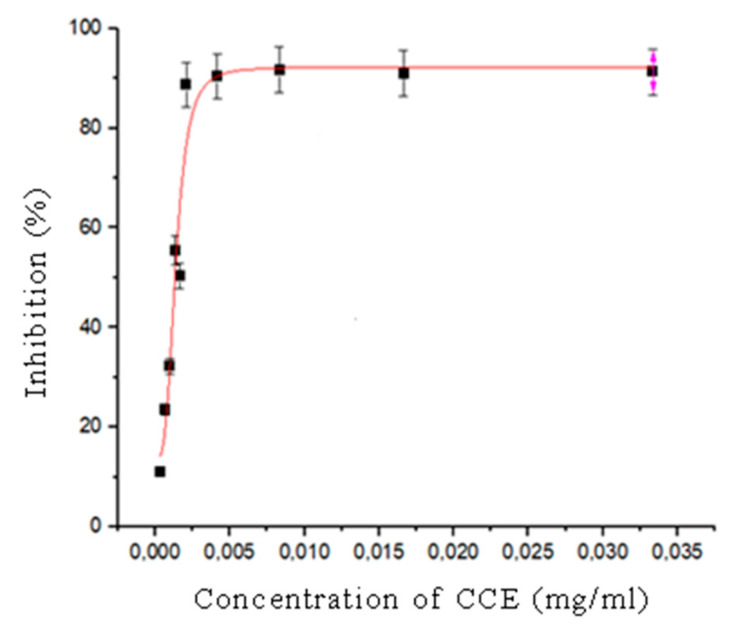
The inhibition-concentration curve of CCE using DPPH assay.

The antioxidant capacity of the CCE was assessed by measuring its DPPH (2,2-diphenyl-1-picrylhydrazyl) free radical scavenging activity [24]. Starting from a concentration of 1.0 µg/mL, different concentrations of aqueous CCE were tested in two separate batches (1.0, 0.5, 0.25, 0.12, and 0.06 mg/mL) and (0.05, 0.04, 0.03, 0.02, and 0.01 mg/mL). The IC_50_ (half-maximal inhibitory concentration) value of CCE was determined to be 0.0014 mg/mL ± 0.0001 (Figure 2), indicating that a very low concentration of CCE is required to achieve significant antioxidant activity. The result highlighted the potent antioxidant properties of CCE, as demonstrated by its ability to significantly reduce free radicals at minimal concentrations. This makes CCE a promising candidate for further studies related to its potential health benefits, particularly in preventing oxidative stress-related damage. Therefore, CCE could potentially be a valuable natural antioxidant in food, pharmaceutical, or cosmetic applications.

### 3.3. Manufacture of Films without CCE Prepared as Function of Different Concentrations of GLY

**Figure 3:**
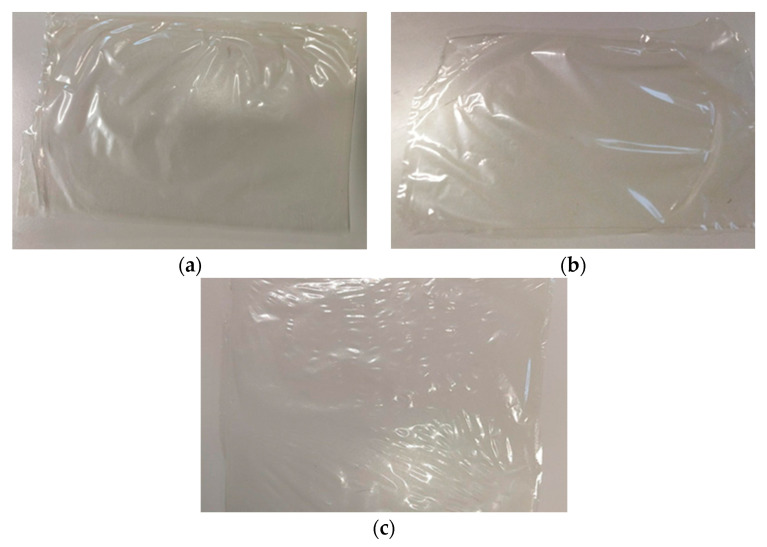
Chitosan (CH)-based films with (**a**) 20% GLY (*w*/*w* of CH); (**b**) 30% GLY (*w*/*w* of CH); (**c**) 50% GLY (*w*/*w* of CH).

As stated by [34], pure CH films are resistant but are too brittle for industrial applications. To confirm and build upon previous studies, in addition to the introduction of the new casting method (e.g., Coatmaster), which lacked existing literature for reference, various films with 2% CH as a polysaccharide source with different percentages of GLY as a plasticizer (0%, 10%, 20%, 30%, and 50% *w*/*w* of CH) were prepared. As expected, the film with 0% GLY could not be studied as it was too fragile, and it was not able to be removed intact from Coatmaster. Furthermore, the film prepared with 10% GLY showed a similar behavior to the previous one. Instead, films with 20%, 30%, and 50% GLY were able to be removed intact and be analyzed (Figure 3) without any microscopic differences.

### 3.4. Characterization of Films

#### 3.4.1. Mechanical Properties

**Figure 4:**
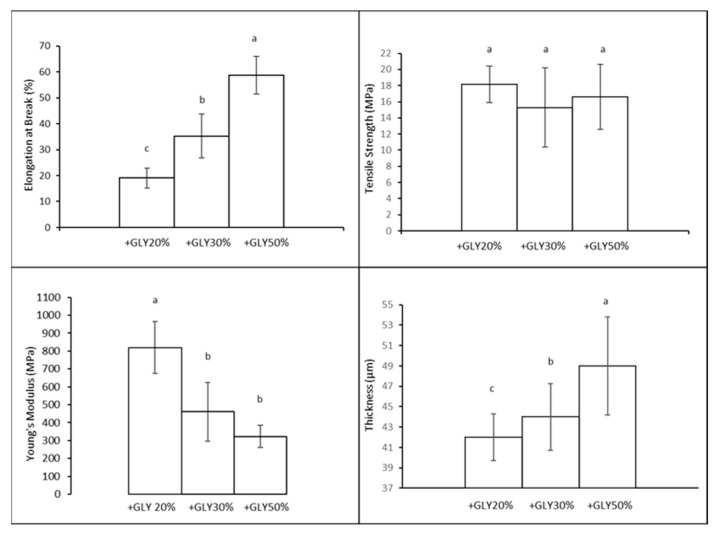
Mechanical properties and thickness of films produced with different concentrations of GLY (*w*/*w* of CH). Different lowercase letters (a–c) denote significant differences among the values reported in each bar (Duncan’s multiple range tests, *p* < 0.05).

In this work, we compared the mechanical properties of CH bioplastics made with different percentages of GLY. In particular, it is worth pointing out that, as expected, the film prepared using 50% GLY seems to exhibit the highest EB (Figure 4). The impact of GLY on EB has been demonstrated quite extensively by numerous researchers [34]. The results of TS and YM agree with each other, showing a lower TS and YM for a higher percentage (30–50%) of GLY films compared to the 20% GLY one; a lower YM indicates a reduced stiffness, while the reduction in TS is due to a poor resistance (Figure 4). For the tested conditions, it is possible to see that with increasing GLY percentage, there is a decrease in the TS and the YM, and this is a classic behavior due to the plasticizer itself.

The study confirmed that adding more glycerol to CH bioplastics made them more flexible but also weaker and less stiff. This trade-off is important to consider when designing bioplastics for specific applications, where the balance between flexibility and strength needs to be carefully managed [35].

#### 3.4.2. Moisture Content and Moisture Uptake

Water is one of the most important plasticizers of biological systems, as water particles form hydrogen bonds with the polymeric chains inside the system. Thus, water impacts the physical properties of biopolymers. The results are summarized in Table 2:

The data from the 30% plasticizer are consistent with those obtained in [36], as the latter obtained a moisture content of low and high molecular weights for CH of 21.57 ± 0.67 and 19.24 ± 0.23, respectively versus the obtained value of 20.19 ± 0.003. Moreover, it was observed that solutions with higher GLY concentrations resulted in films with significantly greater moisture content. Conversely, an increase in GLY presence decreased the moisture uptake of the films. The GLY is likely able to form a barrier that prevents moisture from being absorbed or released too quickly, which is potentially essential for preserving the quality of different food items. Therefore, among these options, the choice of CH film with 30% GLY (*w*/*w* of CH) content was particularly significant for several reasons. CH films with 30% GLY exhibited an optimal balance between flexibility and rigidity and showed good extensibility, an excellent ability to withstand, and a lower stiffness of the films without the need to use an excessive amount of plasticizer. In addition, the choice of 30% GLY content as a plasticizer struck a balance between the film’s mechanical properties and hydrophilicity. When compared to conventional plastics such as LDPE, commonly used in food packaging, CH-based films with 30% GLY present both benefits and challenges. LDPE is known for its excellent flexibility and toughness, which makes it a popular choice for packaging [37]. The 30% GLY CH films achieve similar flexibility but with the added benefit of being biodegradable, unlike LDPE. LDPE is highly effective at preventing moisture loss, which is essential for preserving food quality. Similarly, CH films with 30% GLY also exhibit strong moisture barrier properties, suggesting they could be a viable alternative to LDPE in specific applications. However, while CH films offer the added advantage of being biodegradable, further consideration is needed to fully address their performance compared to LDPE in diverse packaging scenarios [37].

### 3.5. Manufacture of Films with CCE Prepared with 30% of Glycerol (GLY, w/w of CH)

#### 3.5.1. Zeta Size and Zeta Potential of the Film-Forming Solutions (FFSs)

To assess the stability of CCE dissolved in distilled water at distinctive concentrations in CH, the impact of the CCE on the surface charge potential (Z-potential) and particle dimension (Z-size) of FFSs at pH 2 was considered over time. To this end, each FFS was tested with and without different amounts of the CCE (+0%, +1.25%, +2.5%, +5%). The results in Table 3 show the changes at 0, 2, 4, and 6 h.

No significant differences were observed in either FFSs Z-Potential or Z-size at different times. These results indicated that all FFSs were steady. The Z-potential values measured consistently higher than +30 mV, so the solution or dispersion will resist aggregation, which is a key indicator of stability, as it suggests that the particles within the solution carry a sufficient charge to resist aggregation. The obtained results are in line with the ones reported by [38], who studied the surface charge and particle size of chitosan-based film incorporating flower extracts.

Generally, the particle charge is affected by the solvent used [36], since the pH and components can impact the surface charge of dissolved or suspended particles.

It is important to point out that the solution had pH 2, and no other pH ranges were considered as CH would aggregate in the solution. Additionally, the particle dimension decreased with increasing extract concentrations in the diverse FFSs. The polydispersity index (PDI) was demonstrated to be a useful parameter for characterizing the heterogeneity of particle sizes within a solution, with PDI values ranging from 0.5 to 0.7. The samples were considered polydisperse, indicating a notable degree of variety in particle size distribution, with particles of different sizes present.

#### 3.5.2. FT-IR Film

**Figure 5:**
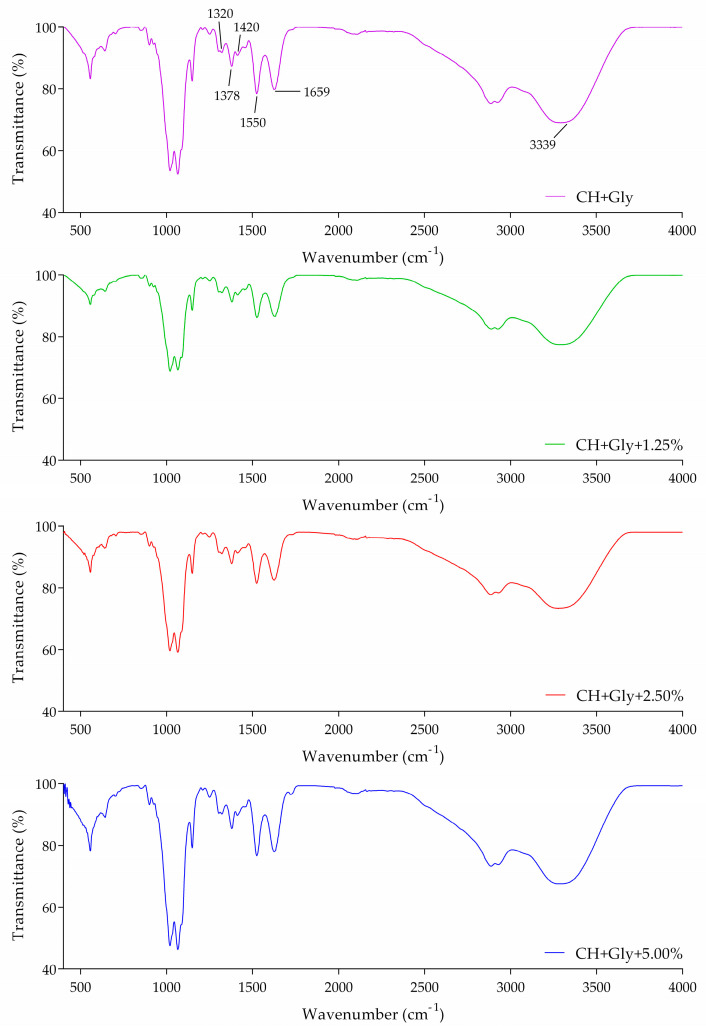
FTIR spectra of chitosan-based films with or without different final concentrations of CCE.

Figure 5 shows the Fourier-transform infrared spectroscopy (FTIR) spectra of films made from chitosan manufactured with glycerol and different percentages of CCE. The chitosan-based films detected characteristic bands at approximately 3339, 1659, 1550, 1320, 1420, and 1378 cm^−1^, which are attributed to the stretching vibration of N-H and O-H groups included in hydrogen bonds, the C=O stretching of amide I, the N-H bending of amide II, the C-N stretching of amide III, CH_2_ bending, and CH_3_ symmetrical deformations, respectively. Furthermore, the presence of an asymmetric stretching band of the C-O-C glycosidic bridge bond band centered around 1154 cm^−1^. The addition of CCE showed minor changes in the region 1500–1700 cm^−1^, ascribed to the characteristic stretching vibration of the ester carbonyl group of flavonoids. The inspection of the spectra also showed a dispersion of compounds present in CCE into the film’s matrix.

#### 3.5.3. Film Mechanical Properties and Sealing Strength

**Figure 6:**
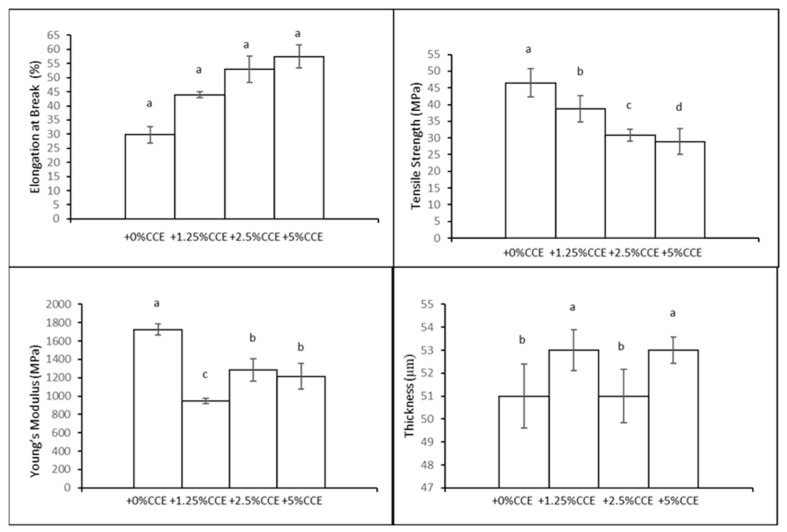
Mechanical properties and thickness of films obtained with different concentrations of *Callistemon citrinus* extract (CCE) (*w*/*w* of CH). Different lowercase letters (a–d) denote significant differences between the values shown in each bar (*p* < 0.05).

To identify any significant alterations in the film’s mechanical properties with the addition of CCE, the mechanical properties were evaluated; namely, TS, EB, and YM were evaluated. The obtained results are described in Figure 6. It is possible to see a homogeneous pattern in the results (Figure 6). In particular, it is possible to notice an increase in EB with the increasing amount of CCE and, at the same time, TS and YM decrease accordingly; the CCE extract acts as a plasticizer with a very good performance. It is possible to see, for example, that adding +5% (*w*/*w* CH) of CCE extract to a CH film with 30% GLY increases its EB (%) to 57.40 ± 4.14, which is comparable to 58.71 ± 7.23 of 50% GLY films. The TS (MPa) value for +5% CCE is 28.91± 3.91 and for YM (MPa) it is 1215.77± 142.76. The corresponding values of TS (MPa) and YM (MPa) for 50% GLY are 16.60 ± 4.01 and 321.97± 61.74, respectively. It is possible to say that +5% CCE makes the film withstand the same elongation as the 50% GLY film and, at the same time, increases both the resistance to breakage and the stiffness.

When compared to conventional low-density polyethylene (LDPE), which has an elongation at break of 349% and a tensile strength of 9.93 MPa [10], the CH films with 5% CCE exhibit much lower elongation but significantly higher tensile strength. While LDPE is highly flexible, as indicated by its high EB, the CH film with CCE offers greater strength and stiffness, making it more resistant to breakage under lower strains. Additionally, the CH films tested had a thickness of around 52 µm, which is thinner than the 85 µm typical of LDPE films, potentially offering material savings and reduced environmental impact. Although LDPE is highly flexible, CH films with 5% CCE offer a stronger and stiffer alternative while still retaining sufficient flexibility. This makes them a promising choice for applications that require enhanced mechanical strength without significantly compromising elongation [39].

The results in Table 4 indicate that the addition of CCE affected the sealing strength. In comparison, LDPE showed a higher value, highlighting the difference between conventional materials and the bioplastics with CCE. Additionally, it was possible to heat seal all the films successfully. However, the films exhibited good compatibility, allowing the formation of secure and lasting seals for an application in the food packaging sector [40].

#### 3.5.4. Film Water Contact Angle (WCA)

It was possible to measure the WCA after 30 s only for the film with the highest percentage (5%) of the extract. In fact, with the increase in the percentage of polyphenols, the films were less hydrophilic (Table 5).

**Figure 7:**
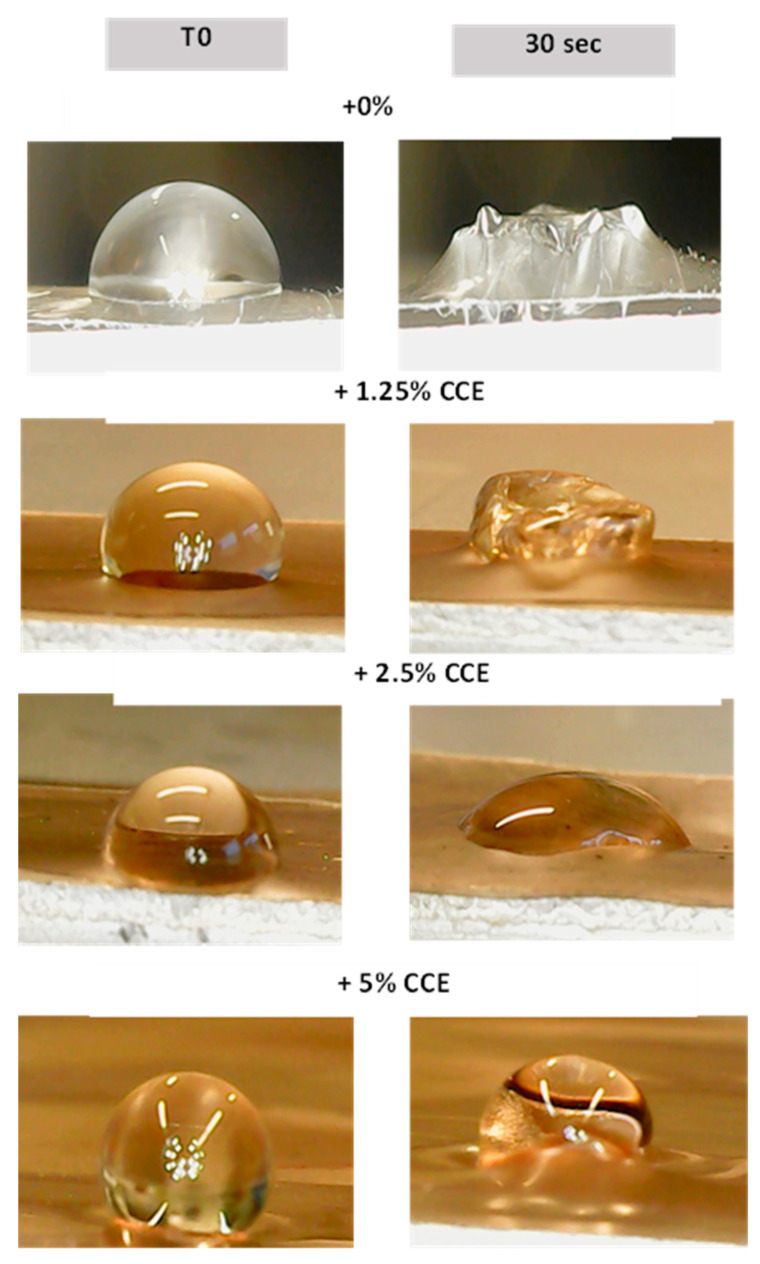
Water contact angle of films recorded at T0 and T30.

The WCA of the films is depicted in Figure 7, where this parameter increases by increasing the concentration of the extract, proving that the additive from the flower can decrease the film surface hydrophilicity. The results indicate that the flower extract effectively reduced the hydrophilicity of the film surface [41,42]. As the concentration of the extract increased, the films became more water-resistant, as evidenced by the higher WCA. This change in surface properties could enhance the film’s performance in applications where moisture resistance is desirable [43].

#### 3.5.5. Film Barrier Properties

The films’ gas barrier properties are evaluated [11] in Table 6. These features are extremely important for industrial applications of bioplastics, particularly in packaging, where controlling the permeability of gases such as CO_2_ and water vapor can significantly affect the product’s shelf life and quality. The films functionalized with the CCE exhibited lower CO_2_ permeability values compared to those measured for low-density polyethylene (LDPE), one of the most common oil-derived plastics. This reduction in CO_2_ permeability could be attributed to structural changes formed following the incorporation of bioactive molecules into the CH matrix, indicating that these films are more effective at preventing the passage of CO_2_ gas. In contrast, the water vapor permeability was notably higher compared to the reference sample LDPE. The decrease in the WV barrier properties of the films containing CCE may be due to the non-uniform particle size distribution in the solution, which could potentially enhance the permeation and migration of water vapor through the film matrix [44]. Thus, while the CCE-functionalized films offer improved CO_2_ resistance, their water vapor barrier properties may need further optimization for applications where moisture control is critical.

#### 3.5.6. Films Antioxidant Activities

**Figure 8:**
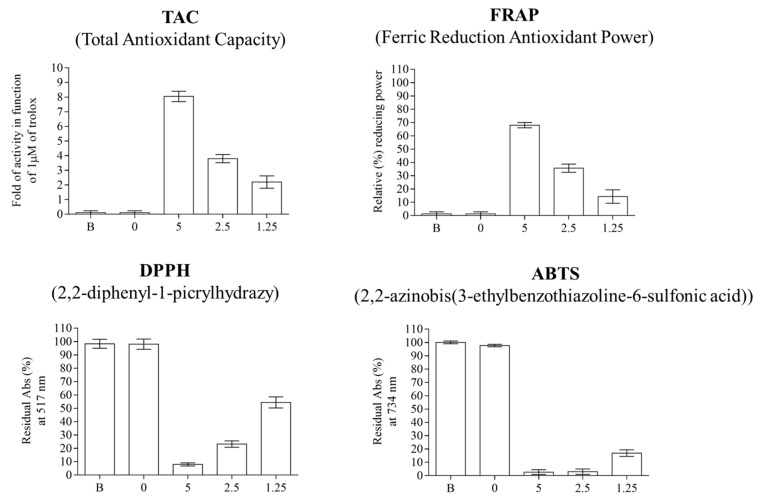
Effect of different percentages of CCE on antioxidant activity of bioplastics prepared with chitosan (CH, 2%) and GLY (30% *w*/*w* of CH) through TAC, FRAP, DPPH, and ABTS assays. (B: internal standard; 0: film control (CH + GLY); 5: film + 5% of CCE; 2.5: film + 2.5% of CCE; 1.25: film + 1.25% of CCE).

To assess the antioxidant capacity of the CCE-enriched films, we conducted standard antioxidant tests, such as 2,2-diphenyl-1-picrylhydrazyl (DPPH), total antioxidant capacity (TAC), ferric ion-reducing antioxidant power (FRAP), and 2,2′-Azino-bis (3-ethylbenzothiazoline-6-sulfonic acid) (ABTS). Starting from 1.0 µg/mL of homogenized aqueous film solutions, the same different concentrations were tested. As can be seen in Figure 8, chitosan-based films alone exhibited no activity in any of the assays performed, while the films exhibited noticeable dose-dependent antioxidant and free-radical scavenging activity due to the presence of CCE. Similar results were observed in the FRAP assay, which demonstrated the films’ capability to convert the ferric ion (Fe^3+^)-ligand complex into the deep blue ferrous (Fe^2+^) complex. Such activity was observed exclusively in CH-based films functionalized with 1.25%, 2.5%, and 5% CCE, revealing a gradual increase in activity. Furthermore, in this case, no activity was reported for the CH-based films without functionalization with CCE (Figure 8). The acquisition of antioxidant activity by CH-based films functionalized with CCE is evident in the total antioxidant capacity assay commonly and routinely used to evaluate the total antioxidant capacity of samples, based on the ability of the tested compounds to change the oxidation state of Cu^2+^ to Cu^+^ following chelation with a colorimetric dye, which shows a maximum absorbance at 570 nm. The analysis of the data showed that the activity is dependent on concentrations, with an activity corresponding to 8.0-, 3.8-, and 2.2-fold of a corresponding solution with a concentration of Trolox of 1 mM. The increase in antioxidant capacity is evident across all tested assays and is directly related to the concentration of CCE in the films. This makes the CCE-enriched chitosan films promising for applications where antioxidant properties are beneficial, such as in food packaging or other protective coatings [45].

## 4. Conclusions

The present study explored chitosan-based bioplastics enriched with an extract from *Callistemon citrinus* (CCE) that, with its bright red color, has incredible antioxidant activity and could be used for the production of reddish films for the packaging and/or food sector, making the product more appealing to consumers. It is worth noting that the presence of CCE keeps the FFS stable, as proved by the zeta potential values, which were consistently above +30 mV with a particle size decreasing over time and set after 6 h, further confirming the stability of the solution.

Through the preparation and characterization of the chitosan-based films enriched with the flower extract, it was possible to obtain 30% GLY+ 5% CCE films with a good technological attitude.

The mechanical properties of the 30% GLY + 5% CCE film showed an EB of 57.40% ± 4.14, which is comparable to the EB of 58.71% ± 7.23 for 50% GLY films. Additionally, the TS and YM for the 5% CCE film were 28.91 ± 3.91 MPa and 1215.77 ± 142.76 MPa, respectively. These results indicate that adding 5% CCE allowed the film to achieve similar elongation to the 50% GLY film while simultaneously enhancing both resistance to breaking and stiffness, demonstrating that CCE may also act as a plasticizer.

The chitosan-based film with 30% GLY, selected for the addition of the flower extract, exhibited a moisture content of 20.19 ± 0.003 and a moisture uptake of 8.98 ± 0.001. These values indicate that GLY acted as a barrier, regulating moisture exchange and helping to preserve food quality. Upon inserting the CCE into the FFS prepared with 30% GLY, it is interesting to note that the materials exhibited a lower CO_2_ permeability, being 7.26 ± 0.5 cm^3^ mm m^−2^ d ^−1^ kPa^−1^ for the neat films and 0.16 ± 0.01 cm^3^ mm m^−2^ d ^−1^ kPa^−1^ for the ones prepared with the highest concentrations of the flower extract. On the other hand, the H_2_O barrier property showed a worsening in the presence of the extract (10.85 ± 0.50 g mm m^−2^ d ^−1^ kPa^−1^ vs. 13.64 ± 0.52 g mm m^−2^ d ^−1^ kPa^−1^) even though the 30% GLY+ 5% CCE films showed increased film surface hydrophobicity, as investigated by contact angle measurements.

Furthermore, the antioxidant properties of the flower extract stayed intact and did not lose any activity when mixed into the chitosan-based films.

In conclusion, this work takes the first step toward a deeper understanding and use of flower extracts in the food packaging sector.

## Figures and Tables

**Table 1:** Identification and evaluation of composites in CCE were performed using RP-HPLC-DAD-ESI-MS/MS investigation. The results are shown as the average ± S.D. from three isolated tests, with the amounts of the composites expressed as mg of cyanidin-3-*O*-glucoside coequals (CyG/100 g of dry extract).

Rt	Compounds	Fragmentation Pattern	Quantification
17.8	Cyanidin 3,5-*O*-diglucoside	MS:611; MS:611, 449, 287	255.27 ± 2.39
20.4	Peonidin-3,5-*O*-diglucoside	MS:625; MS:625, 463, 301	187.22 ± 2.35
22.7	Cyanidin-3-*O*-glucoside	MS:449; MS:449, 287	33.13 ± 0.85
25.1	Cyanidin-coumaroylglucoside-pyruvic acid	MS:661; MS:661, 595, 482	10.81 ± 0.78

**Table 2:** Moisture content and moisture uptake of CH-based films with different percentages of glycerol (20%, 30%, 50% *w*/*w* of CH).

	Moisture Content (%)	Moisture Uptake (%)
+20% GLY	16.57 ± 0.002 ^c^	12.44 ± 0.002 ^a^
+30% GLY	20.19 ± 0.003 ^b^	8.98 ± 0.001 ^b^
+50% GLY	39.90 ± 0.009 ^a^	6.98 ± 0.001 ^c^

Diverse small letters (a–c) demonstrate critical differences among the values detailed in each column (Duncan’s multiple range tests, *p* < 0.05).

**Table 3:** The surface charge potential (Z-Potential) and particle size distribution (Z-size) of the different FFSs recorded at time 0 (T0) and after 2 (T2), 4 (T4) and 6 (T6) hours from their preparation. PDI, polydispersity index.

**T0**	**Z-Size (d.nm)**	**Z-Potential (mV)**	**PDI**
FFS + 0% CCE	1239 ±166 ^a^	36.2 ± 3.1 ^a^	0.52 ± 0.02 ^a^
FFS + 1.25% CCE	1221 ± 91 ^a^	35.0 ± 0.4 ^a^	0.47 ± 0.04 ^b^
FFS + 2.5% CCE	995 ± 12 ^b^	35.7 ± 1.9 ^a^	0.54 ± 0.01 ^a,b^
FFS + 5% CCE	992 ± 33 ^b^	40.1 ± 3.7 ^a^	0.53 ± 0.02 ^a,b^
**T2**	**Z-Size (d.nm)**	**Z-Potential (mV)**	**PDI**
FFS + 0% CCE	1189 ± 96 ^a^	41.6 ± 5.0 ^a^	0.73 ± 0.06 ^b,c^
FFS + 1.25% CCE	985 ± 192 ^a,b^	34.7 ± 3.9 ^b^	0.72 ± 0.10 ^b^
FFS + 2.5% CCE	758 ± 250 ^b^	42.1 ± 1.6 ^a^	0.55 ± 0.01 ^a^
FFS + 5% CCE	754 ± 87 ^b^	41.7 ± 0.6 ^a^	0.67 ± 0.05 ^c^
**T4**	**Z-Size (d.nm)**	**Z-Potential (mV)**	**PDI**
FFS + 0% CCE	1060 ± 47 ^a^	45.2 ± 2.6 ^a,b^	0.65 ± 0.12 ^a^
FFS + 1.25% CCE	851 ± 107 ^b^	33.5 ± 18.0 ^c^	0.61 ± 0.06 ^b^
FFS + 2.5% CCE	828 ± 112 ^b^	40.3 ± 4.9 ^b^	0.69 ± 0.09 ^a,b^
FFS + 5% CCE	810 ± 6 ^b^	46.1 ± 0.9 ^a^	0.64 ± 0.13 ^a,b^
**T6**	**Z-Size (d.nm)**	**Z-Potential (mV)**	**PDI**
FFS + 0% CCE	954 ± 52 ^a,b^	40.3 ± 5.7 ^a^	0.52 ± 0.03 ^a^
FFS + 1.25% CCE	1069 ± 43 ^a^	39.9 ± 2.0 ^a^	0.59 ± 0.03 ^a^
FFS + 2.5% CCE	886 ± 57 ^b,c^	41.3 ± 4.4 ^a^	0.57 ± 0.01 ^a^
FFS + 5% CCE	772 ± 98 ^c^	46.4 ± 3.2 ^a^	0.56 ± 0.06 ^a^

Different small letters (a–c) show significant differences among the values reported in each column (Duncan’s multiple range tests, *p* < 0.05). Advance test elements are given in text.

**Table 4:** Sealing strength value of the chitosan-based films functionalized with CCE.

Film	Sealing Strength (N/cm)
+0% CCE	0.82 ± 0.04 ^a^
+1.25% CCE	0.73 ± 0.03 ^b^
+2.5% CCE	0.73 ± 0.05 ^b^
+5% CCE	1.20 ± 0.1 ^a^
LDPE	5.35 ± 0.13 ^c^

Lowercase letters (a–c) represent significant variations between the values listed in each column (*p* < 0.05).

**Table 5:** Water contact angle measurement of the functionalized films.

Film Type	0 s	30 s
+0% CCE	90.48 ± 3.4 ^b^	n.a.
+1.25% CCE	106.3 ± 4.3 ^a^	n.a.
+2.5% CCE	86.52 ± 3.7 ^b^	n.a.
+5% CCE	114.80 ± 14.3 ^a^	101.22 ± 7.3 ^a^

Distinct letters (a,b) represent significant variations between the values listed in each column (*p* < 0.05).

**Table 6:** Gas barrier properties of the prepared films. LD-PE (low-density polyethylene) was used as a reference material.

	CO_2_ (cm^3^ mm m^−2^ d ^−1^ kPa^−1^)	WVP (g mm m^−2^ d ^−1^ kPa^−1^)
LDPE	14.09 ± 0.05 ^a^	0.06 ± 0.01 ^b^
+0% CCE	7.26 ± 0.5 ^b^	10.85 ± 0.50 ^b^
+1.25% CCE	0.57 ± 0.01 ^c^	14.09 ± 0.61 ^a^
+2.5% CCE	0.27 ± 0.02 ^d^	16.01 ± 0.40 ^a^
+5% CCE	0.16 ± 0.01 ^d^	13.64 ± 0.52 ^a^

Different lowercase letters (a–d) signify statistically significant differences between the values listed in each column (Duncan’s multiple range test, *p* < 0.05).

## Data Availability

The original contributions presented in the study are included in the article, further inquiries can be directed to the corresponding author.

## References

[1] Reddy R.L., Reddy V.S., Gupta G.A. (2013). Study of Bioplastics as Green and Sustainable Alternative to Plastics. Int. J. Emerg. Technol. Adv. Eng..

[2] Farajinejad Z., Karimi Sani I., Alizadeh M., Amiri S. (2024). A Review of Recent Advances in the Photocatalytic Activity of Protein and Polysaccharide-Based Nanocomposite Packaging Films: Antimicrobial, Antioxidant, Mechanical, and Strength Properties. J. Polym. Environ..

[3] MacLeod M., Arp H.P.H., Tekman M.B., Jahnke A. (2021). The global threat from plastic pollution. Science.

[4] Zhao X., Cornish K., Vodovotz Y. (2020). Narrowing the Gap for Bioplastic Use in Food Packaging: An update. Environ. Sci. Technol..

[5] Dintcheva N.T., Morici E. (2023). Recovery of rose flower waste to formulate eco-friendly biopolymer packaging films. Molecules.

[6] Rachtanapun P., Klunklin W., Jantrawut P., Jantanasakulwong K., Phimolsiripol Y., Seesuriyachan P., Leksawasdi N., Chaiyaso T., Ruksiriwanich W., Phongthai S. (2021). Characterization of chitosan film incorporated with curcumin extract. Polymers.

[7] Xie C., Wang F., He Z., Tang H., Li H., Hou J., Liu Y., Jiang L. (2023). Development and characterization of active packaging based on chitosan/chitin nanofibers incorporated with scallion flower extract and its preservation in fresh-cut bananas. Int. J. Biol. Macromol..

[8] Dìaz-Montes E., Castro-Munoz R. (2021). Edible films and coating as food-quality preserves: An overview. Foods.

[9] Jorda J.J., Casem D.T., Bradley J.M., Dwivedi A.K., Brown E.N., Jordan C.W. (2016). Mechanical properties of low density polyethylene. J. Dyn. Behav. Mater..

[10] Shebani A., Klash A., Elhabishi R., Abdsalam S., Elbreki H., Elhrari W. (2018). The influence of LDPE content on the mechanical properties of HDPE/LDPE blends. Res. Dev. Mater. Sci..

[11] Phan The D., Debeaufort F., Voilley A., Luu D. (2009). Biopolymer interactions affect the functional properties of edible films based on agar, cassava starch and arabinoxylan blends. J. Food Eng..

[12] Fiallos-Nunez J., Cardero Y., Cabrera-Barjas G., Garcìa-Herrera C.M., Inostroza M., Estevez M., Espana-Sànchez B.L., Valenzuela L.M. (2024). Eco-friendly design of chitosan-based films with biodegradable properties as an alternative to low-density polyethylene packaging. Polymers.

[13] Xuan Tan S., Andriyana A., Chyuan Ong H., Lim S., Ling Pang Y., Cheng Ngoh G. (2022). A comprehensive review on the emerging roles of nanofillers and plasticizers towards sustainable starch-based bioplastics fabrication. Polymers.

[14] Ling Tan Y., Peng Teoh Y., Xian Ooi Z., Hoong Shuit S., Hwa Ng Q., Yong Hoo P., Siong Leong S., Yu Low C. (2023). Effect of glycerol as Plasticizing Agent on the Mechanical Properties of Polyvinyl Alcohol/Banana Peel Powder Blended Film. Emerg. Technol. Future Sustain..

[15] Nechita P., Roman M., Nastac S.M. (2023). Green approaches on modification of xylene hemicellulose to enhance the functional properties for food packaging materials—A review. Polymers.

[16] Sanyang M.L., Sapuan M.S., Jawaid M., Ishak M.R., Sahari J. (2015). Effect of plasticizer type and concentration on tensile, thermal and barrier properties of biodegradable films based on sugar palm (*Arenga pinnata*) starch. Polymers.

[17] Suyatma N.E., Tighzert L., Copinet A. (2005). Effect of hydrophilic plasticizers on mechanical, thermal and surface properties of chitosan films. J. Agric. Food Chem..

[18] Calva-Estrada S., Jimenez M., Lugo E. (2019). Protein-Based Films: Advances in the Development of Biomaterials Applicable to Food Packaging. Food Eng. Rev..

[19] Abourehab M.A.S., Pramanik S., Abdelgawad M.A., Abualsoud B.M., Kadu A., Ansari M.J., Deepak A. (2022). Recent Advances of Chitosan Formulations in Biomedical Applications. Int. J. Mol. Sci..

[20] Fayemi P.O., Öztürk I., Özcan C., Muguruma M., Yetim H., Sakata R., Ahhmed A. (2017). Antimicrobial activity of extracts of *Callistemon citrinus* flowers and leaves against Listeria monocytogenes in beef burger. J. Food Meas. Charact..

[21] Laganà G., Barreca D., Smeriglio A., Germanò M.P., D’Angelo V., Calderaro A., Bellocco E., Trombetta D. (2020). Evaluation of Anthocyanin Profile, Antioxidant, Cytoprotective, and Anti-Angiogenic Properties of *Callistemon citrinus* Flowers. Plants.

[22] Altemini A., Lakhssassi N., Baharlouei A., Watson D.G., Lightfoot D.A. (2017). Phytochemicals: Extraction, isolation and identification of bioactive compounds from plant extracts. Plants.

[23] Singleton V.L., Orthofer R., Lamuela-Raventós R.M. (1999). [14] Analysis of total phenols and other oxidation substrates and antioxidants by means of Folin-Ciocalteu reagent. Methods Enzymol..

[24] Lesjak M., Beara I., Simin N., Pintać D., Majkić T., Bekvalac K., Orčić D., Mimica-Dukić N. (2018). Antioxidant and anti-inflammatory activities of quercetin and its derivatives. J. Funct. Foods..

[25] Mirpoor S.F., Giosafatto C.V.L., Di Girolamo R., Famiglietti M., Porta R. (2022). Hemp (*Cannabis sativa*) seed oilcake as a promising by-product for developing protein-based films: Effect of transglutaminase-induced crosslinking. Food Packag. Shelf Life.

[26] (1997). Standard Test Method for Tensile Properties of Thin Plastic Sheeting.

[27] Giosafatto C.V.L., Di Pierro P., Gunning P., Mackie A., Porta R., Mariniello L. (2014). Characterization of Citrus pectin edible films containing transglutaminase-modified phaseolin. Carbohydr. Polym..

[28] Al-Asmar A., Giosafatto C.V.L., Sabbah M., Sanchez A., Villalonga Santana R., Mariniello L. (2020). Effect of Mesoporous Silica Nanoparticles on The Physicochemical Properties of Pectin Packaging Material for Strawberry Wrapping. Nanomaterials.

[29] Mirpoor S.F., Zannini D., Santagata G., Giosafatto C.V.L. (2024). Cardoon seed oil cake proteins ad substrate for microbial transglutaminase: Their application as matrix for bio-based packaging to extend the shelf-life of peanuts. Food Hydrocoll..

[30] (2010). Standard Test Method for Oxygen Gas Transmission Rate Through Plastic Film and Sheeting Using a Coulometric Sensor.

[31] (2005). Standard Test Method for the Determination of Carbon Dioxide Gas Transmission Rate Through Barrier Materials Using an Infrared Detector.

[32] (2013). Standard Test Method for Water Vapor Transmission Rate Through Plastic Film and Sheeting Using a Modulated Infrared Sensor.

[33] Barreca D., Laganà G., Leuzzi U., Smeriglio A., Trombetta D., Bellocco E. (2016). Evaluation of the nutraceutical, antioxidant and cytoprotective properties of ripe pistachio (*Pistacia vera* L., variety Bronte) hulls. Food Chem..

[34] Farahnaky A., Saberi B., Majzoobi M. (2013). Effect of Glycerol on Physical and Mechanical Properties of Wheat Starch Edible Films. J. Texture Stud..

[35] Tan X.T., Ong H.C., Andriyana A., Lim S., Pang Y.L., Kusumo F., Ngoh G.C. (2022). Characterization and parametric study on mechanical properties enhancement in biodegradable chitosan-reinforced starch-based bioplastic film. Polymers.

[36] Bhattacharjee S. (2016). DLS and zeta potential—What they are and what they are not?. J. Control. Release.

[37] Wronska N., Katir N., Nowak-Lange M., El Kadib A., Lisowska K. (2023). Biodegradable chitosan-based films as an alternative to plastic packaging. Foods.

[38] Yang Y. (2024). Development of chitosan-based films incorporated with chestnut flower essential oil that possess good anti-ultraviolet radiation and antibacterial effects for banana storage. Coatings.

[39] Lòpez-Mata M.A., Ruiz-Cruz S., Ornelas-Paz J.J., Del Toro-Sànchez C.L., Màrquez-Rìos E., Silva-Beltràn N.P., Cira-Chàvez L.A., Burruel-Ibarra S.E. (2018). Mechanical, barrier and antioxidant properties of chitosan films incorporating cinnamaldehyde. J. Polym. Environ..

[40] Azanha de Carvalho F., Moreira A.A., Martinez de Oliveria A.L., Yamashita F. (2021). Biodegradation of poly(lactic acid)-cassava bagasse composites produced by injection molding. J. Appl. Polym. Sci..

[41] Prus-Walendziak W., Kozlowska J. (2021). Design of sodium alginate/gelatin-based emulsion film fused with polylactide microparticles charged with plant extract. Materials.

[42] Goudar N., Vanjeri V.N., Masti S.P., Chougale R.B. (2020). *Spathodea campanulata* bud fluid reinforced mechanical, hydrophilicity and degradation studies of poly (vinyl alcohol) matrix. Appl. Sci..

[43] Raza Z.A., Khatoon R., Banat I.M. (2021). Altering the Hydrophobic/Hydrophilic Nature of Bioplastic Surfaces for Biomedical Applications. Bioplastics Sustain. Dev..

[44] Laboulfie F., Hèmati M., Lamure A., Diguet S. (2013). Effect of the plasticizer on permeability, mechanical resistance and thermal behavior of composite coating films. Powder Technol..

[45] Subramani G., Manian R. (2024). Bioactive chitosan films: Integrating antibacterial, antioxidant, and antifungal properties in food packaging. Int. J. Biol. Macromol..

